# Awareness, treatment, control of hypertension and utilization of health care services following screening in the North-central region of Burkina Faso

**DOI:** 10.11604/pamj.2014.19.259.4707

**Published:** 2014-11-08

**Authors:** Boukaré Doulougou, Séni Kouanda, Gautier Henri Ouédraogo, Bertrand Ivlabèhiré Meda, Aristide Bado, Maria Victoria Zunzunegui

**Affiliations:** 1 Institut de Recherche en Sciences de la Santé (IRSS), 03 BP 7192 Ouagadougou 03, Burkina Faso; 2 Département de Médecine Sociale et Préventive, École de Santé Publique, Université de Montréal, Montréal, QC, Canada; 3 Institut de Recherche en Santé Publique de l'Université de Montréal (IRSPUM), Montréal, QC, Canada; 4 Centre de Recherche du Centre Hospitalier de l'Université de Montréal (CRCHUM), Montréal, QC, Canada

**Keywords:** Awareness, treatment, control, hypertension, Burkina Faso, follow up study

## Abstract

**Introduction:**

In Africa, a non-urban area is affected by hypertension. But in Burkina Faso, no study on factors associated with awareness, treatment and control of hypertension has not yet been published. The objectives of this report are to: (i) identify the factors associated with awareness, treatment, and control of hypertension in the adult population of Kaya health and demographic surveillance system (Kaya HDSS) and (ii) estimate health care services utilization by participant newly screened as hypertensive.

**Methods:**

A screening survey for hypertension was conducted on 1481 adults in Kaya HDSS in late 2012. Hypertensive individuals provided information relating to “awareness”, “treatment” and “control” of their hypertension. After approximately two months, unaware hypertensive individuals were interviewed to know whether they had sought treatment.

**Results:**

During the screening survey, 123 individuals (9.4%) were identified as having hypertension. Among them, 33 (26.8%, 95% CI: 18.9-34.8) were aware of their condition, 25 (75.8%, 95% CI: 60.3-91.2) of them were receiving medication. Among those receiving treatment, 15 (60.0%, 95% CI: 39.4-80.6) had their blood pressure controlled. Semi-urban residence, presence of chronic diseases and physical inactivity were significantly associated with awareness of hypertension. Seventy two of the 90 participants who were classified as unaware were interviewed two months later. Out of them, 37 individuals had consulted a health worker and 28 received a diagnosis of hypertension.

**Conclusion:**

Awareness was low but treatment and control of those who knew they were hypertensive were relatively high. These results could be used to improve management of hypertension in Burkina Faso.

## Introduction

Of the 57 million estimated deaths in 2008, cardiovascular diseases were responsible for 17 million deaths, of these, 80% occurred in low and middle-income countries [1]. Hypertension is recognized as a major risk factor of cardiovascular disease [2]. In 2010 it was identified as the primary risk factor for global burden of disease, with 7% of global disability-adjusted life years (DALYs) [3]. Nearly a quarter of the world´s adult population had hypertension in 2000 and an increase of 60% was forecast for 2025. At the same time, in sub-Saharan Africa (SSA) a faster increase was projected [4]. This increasing trend was confirmed by another study, estimating 75 million people with hypertension in SSA in 2008 and 125 million in 2025 [5].

In addition to this projected increase, levels of awareness, treatment and control of hypertension are low in Africa countries. Awareness varied between 15% and 40% [6–8] and the proportion who were treated varied between 14% and 82% [7–9]. Factors associated with better awareness or treatment included older age, being a woman, urban areas of residence and high body mass index (BMI). Control of treated hypertensive did not exceed 40% [8, 10]. Prevalence in urban areas of Burkina Faso has been estimated to be over 20% [11, 12]. We have recently reported a prevalence of 9.4% in the rural and semi-urban areas of Kaya health and demographic surveillance system (Kaya HDSS) [13]. However, estimates of awareness, treatment and control of hypertension are not yet available. Thus, we conducted this investigation to: (i) identify the factors associated with awareness, treatment, and control of hypertension in the adult population of the rural and semi-urban area of the Kaya HDSS; and (ii) estimate health care services utilization by participant newly screened as hypertensive. We anticipate that information from this two-stage study would contribute to strengthen the detection and control of this impending epidemic of hypertension in developing countries.

## Methods

### First stage, prevalence study

A prevalence study of hypertension was conducted in the adult population of Kaya HDSS in Burkina Faso between September and December 2012. Kaya HDSS consists of monitoring demographic and health characteristics of the population of Kaya health district. More detailed description of the site was described elsewhere [14]. The list of households at the Kaya HDSS provided a sampling frame for a rural and a semi-urban stratum. In each household, one adult (aged 18 years and over) was randomly selected and invited to participate.

Information on sex, age, place of residence, marital status, education, and occupation were gathered. Relevant behavioral pattern were queried using the World Health Organization stepwise approach to chronic disease risk factor surveillance (STEPS) instrument for non-communicable disease risk factors [15]. The question “Do you currently smoke tobacco products such as cigarettes, cigars or pipe” was asked. When the answer was negative, a second question “In the past, did you smoke daily” was asked to identify ex-smokers. Smokers and ex-smokers were combined into one group because number of ex-smokers was small. For alcohol, the question “Have you ever consumed alcoholic beverages such as beer, wine, liquor, local beer” was asked. For physical activities, the participants were asked whether they had intensive activities (activities that required a substantial increase in breathing or heart rate for at least 10 minutes) or moderate activities (activity that required moderate increase in breathing or heart rate for at least 10 minutes) in their current occupation. Further, the participants were asked whether they took part in other strenuous activities or moderate activities in their leisure time. Participants who had strenuous or moderate activities were classified as “having vigorous physical activity”; otherwise as “having non-vigorous physical activity”.

For chronic conditions, the participants were asked whether they had ever been informed by a health professional that they had a chronic disease such as diabetes mellitus, cancer, AIDS, heart problems, or asthma. Family history of hypertension was assessed by asking the participants whether one of their relatives including grandparents, father, mother, brother or sister had hypertension. The experience of Blood Pressure (BP) measurement was assessed by asking the question “Is a doctor or health worker has already measured your BP” In case of an affirmative answer, a second question" When was the last measure of your blood pressure" was asked. Weight was measured using a digital scale, with participants lightly dressed and after removal of shoes and pocket contents. Height was measured with participants in standing position without shoes, using a wooden stadiometer. BMI was computed by dividing the weight (kg) with the square of the height (m^2^) and participants were considered “underweight” when their BMI is below 18.5; “normal weight” when BMI is above or equal to 18.5 and below 25; “overweight” when BMI is above or equal 25 and below 30; and “obese” when BMI is above or equal to 30 [16].

Blood pressure (BP) was measured on one occasion at home by trained interviewers. They used a digital automatic sphygmomanometer (Omron 3) with the proper cuff size. At the end of the interview, after about 25 to 30 minutes rest in a sitting position, BP was measured successively on the two arms; on the arm with the higher reading, two other readings were taken one minute apart. The mean of these two readings was used in statistical analysis. Participant who had a systolic BP ≥140 mm Hg and/or diastolic BP ≥90 mm Hg or reported current use of anti-hypertensive medications was considered hypertensive [17].

Three dependent variables concerning a participant's relation to hypertension were assessed.*Awareness*: Participants were considered “aware” of hypertension when they reported having received a diagnosis of hypertension by a professional health care prior to the prevalence survey.*Treatment*: previously diagnosed individuals who reported receiving prescribed pharmacological treatment for hypertension at the time of the interview were considered having “treatment”. Treated hypertensive individuals were asked to show their drug boxes to the interviewer and drugs were recorded. *Control*: a treated hypertensive individuals who had a systolic blood pressure < 140 mm Hg and diastolic blood pressure < 90 mm Hg was considered having the hypertension “controlled”. Non-pharmacological treatments were defined as behavioral advice received from a health care professional for management of hypertension [18]. Participants were asked if they received advice to reduce salt consumption, weight loss, smoking cessation and to reduce alcohol consumption as well as to increase physical activities. Use of traditional medicine to treat hypertension also was ascertained.

### Second stage, follow up interviews

Participants of the follow up interview were hypertensive individuals who were unaware of their hypertension in the first stage. They were advised to seek professional health care for further hypertensive management. After approximately six weeks to two months, interviewers returned to the home of this new hypertensive and conducted an interview using a structured questionnaire. Participants were asked if they had since seen a health professional. If not, they were asked about the reasons for not doing so. If they had done so, they were asked about the frequency they had blood pressure taken and if hypertension was confirmed, the prescribed treatment. No intervention was provided during this follow-up interview. Participants were confirmed as having high blood pressure if they had an average systolic BP ≥ 140 mm Hg and / or average diastolic BP ≥ 90 mm Hg after at least two visits to the health center. Treated new-hypertensive individuals were asked to show their drug boxes to the interviewer.

### Statistical analysis

Proportions of hypertensive participants who were aware of their status, were receiving pharmacological treatment and whose hypertension was controlled were calculated. Chi-square tests were used to compare the proportions of hypertensive participants who were aware of their condition by selected characteristics. Logistic regression analyses were used to compute odds ratios with 95% confidence intervals for selected factors potentially associated with awareness. A significance level of P < .05 was used. For the follow-up study, proportions of people with confirmed diagnosis and prescribed antihypertensive medication were estimated. All statistical analyses were performed using IBM SPSS 20 for Windows.

### Ethics

The study was approved by the Ethics Committee of Health Research at the University of Montreal in Canada and the Ethics Committee on Health Research in Burkina Faso. All participants were free to participate and a written informed consent was obtained.

## Results

**Figure 1:**
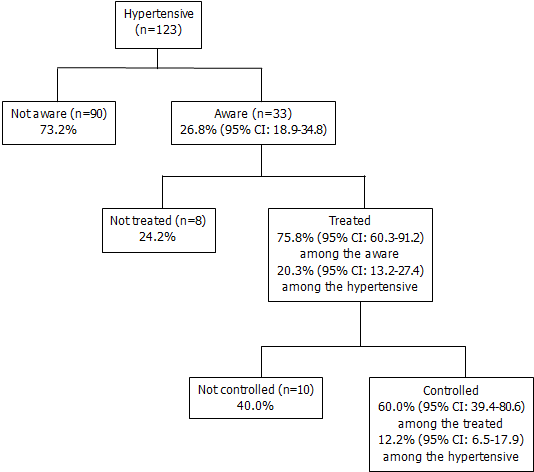
Awareness, treatment and control of hypertension in Kaya HDSS adult population, Burkina Faso

As the first stage survey, 1481 participants have been screened. Among them, only 20% (300 participants) have had their BP measured during the 12 months preceding the survey and 41% have never had their BP measured. After adjustment, women, semi-urban residence, read/write capacity, occupational status of employed and overweight (BMI ≥25) were associated with recent experience of BP measuring (Table 1). Among all the participants, 123 individuals were classified as hypertensive. Thirty three of these hypertensive persons were already aware of their condition and 90 were not.Figure 1 shows the proportion of hypertensive participants who were aware of their condition at the time of the interview, the proportion of hypertensive that reported receiving pharmacological treatment and the proportion of hypertensive who had controlled BP.

**Table 1:** Adjusted odds ratios for experience of blood pressure measuring in the last 12 months

		Blood pressure measurement in the last 12 months		
Variable	N	%	OR (95% CI) unadjusted	OR(95% CI) adjusted
**Sex**				
Men	666	10.8	1	1
Women	815	28.0	3.20 (2.40-4.28)***	3.88 (2.77-5.43)***
**Age (years)**				
18-34	716	22.6	1	1
35-54	516	17.8	0.74 (0.56-0.99)*	0.89 (0.65-1.23)
55 +	249	18.5	0.78 (0.54-1.12)	1.09 (0.70-1.70)
**Residence**				
Rural	726	13.1	1	1
Semi-urban	755	27.2	2.48 (1.89-3.24)***	1.85 (1.35-2.53)***
**Education**				
Illiterate	1056	16.7	1	1
Read/write	425	29.2	2.06 (1.58-2.68)***	1.55 (1.09-2.21)*
**Civil status**				
Married	1224	20.0	1	1
Other	257	21.4	1.09 (0.78-1.51)	0.87 (0.59-1.28
**Occupational status**				
Unpaid job	559	19.7	1	1
Independent	835	17.7	0.88 (0.67-1.16)	1.31 (0.95-1.81)
Employed	87	48.3	3.81 (2.38-6.09)***	4.01 (2.23-7.22)***
**Family history of hypertension**				
No	1215	18.9	1	1
Yes	266	26.3	1.53 (1.12-2.08)**	1.01 (0.70-1.44)
**Chronic conditions**				
No	1429	19.7	1	1
Yes	52	34.6	2.15 (1.20-3.87)*	1.90 (0.96-3.75)
**Body Mass Index^a^**				
< 18.5	227	15.4	0.87 (0.59-1.30)	0.95 (0.62-1.45)
18.5-24.9	996	17.3	1	1
≥ 25.0	194	37.1	2.83 (2.02-3.95)***	1.68 (1.16-2.43)**

a Excluding pregnant women (64)

* *p* < 0.05

** *p* < 0.01

*** *p* < 0.001

Factors associated with awareness are presented in Table 2. Higher odds of awareness were associated with semi-urban area residency (OR = 4.8, 95% CI: 1.6-14.9); having any chronic disease (OR = 8.1, 95% CI: 2.3-28.5) and less than vigorous activity (OR = 3.2, 95% CI: 1.3-7.6). BMI, smoking and alcohol intake were not associated to awareness (results not showed). Among the hypertensive participants who were aware of their condition, three quarters (75.8%, 95% CI: 60.3%-91.2%) were receiving pharmacological treatment. All showed their medicine boxes to the interviewer. Among treated participants, 64% received monotherapy, 32% received bitherapy and only one patient received tritherapy. Calcium channel blockers (CCB) were the most frequently prescribed as monotherapy. In combination therapy, combinations including an Angiotensin Converting Enzyme inhibitor (ACEI) were the most common (Table 3).

**Table 2:** Awareness of hypertension among Kaya HDSS adult hypertensive population, Burkina Faso (n=123)

Variable	n	Aware		
		%	*p-* Value	Odd Ratios (95% CI)
**Sex**				
Male	58	20.7	.15	1.0
Female	65	32.3		1.83 (0.81-4.16)
**Age, y**				
18-34	17	23.5	.45	1.0
35-54	54	22.2		0.93 (0.26-3.38)
55 +	52	32.7		1.58 (0.45-5.57)
**Residence**				
Rural	40	10.0	.003	1.0
Semi-urban	83	34.9		4.83 (1.57-14.92)**
**Civil status**				
Married	91	24.2	.26	1.0
Other	32	34.4		1.64 (0.69-3.93)
**Education**				
Illiterate	87	23.0	.14	1.0
Read/Write	36	36.1		1.89 (0.81-4.40)
**Occupational status**				
Unpaid job	54	31.5	.11	1.0
Independent	61	19.7		0.53 (0.23-1.25)
Employed	8	50		2.18 (0.49-9.76)
**Family history of hypertension**				
No	89	24.7	.39	1.0
Yes	34	32.4		1.46 (0.61-3.46)
**Chronic conditions**				
No	110	21.8	< .001	1.0
Yes	13	69.2		8.06 (2.28-28.47)**
**Physical activity**				
Vigorous	58	15.5	.007	1.0
Non -vigorous	65	36.9		3.19 (1.33-7.62)**

** *p* < 0.01

**Table 3:** The use of various antihypertensive drugs (n=25) by Kaya HDSS hypertensive adult population

Type of drug	n (%)
***One drug*** **(n=16)**	
Calcium antagonists	8 (50.0)
ACEI	3 (18.8)
Diuretics	2 (12.5)
β-blockers	2 (12.5)
Centrally acting sympatholytic	1 (6.3)
***Two drugs*** **(n=8)**	
ACEI + diuretic	3 (37.5)
ACEI + Calcium antagonist	3 (37.5)
Diuretic + Calcium antagonist	1 (12.5)
β-blockers + Calcium antagonist	1 (12.5)
***Three drugs*** **(n=1)**	
ACEI + Calcium antagonist + Diuretic	1 (100)

Abbreviation: ACEI, Angiotensin converting enzyme inhibitor

Among those aware of their hypertension, 28 out of 33 subjects (84.9%, 95% CI: 71.9%-97.8%) received at least one advice as a non-pharmaceutical treatment while among those treated with medication, they were 23 out of 25 subjects (92.0%, 95% CI: 80.6%-100%) (Table 4). Reducing salt consumption was the most frequent advice given (96.4%) to those aware of their hypertension or to patients under pharmacological treatment (95.7%). The proportion of individuals aware of their hypertension who reported having consulted a traditional healer for their hypertension was 21.2% (95% CI: 6.5%-35.9%). Of these participants, one acknowledged having received traditional anti-hypertensive treatment. Among those aware of their hypertension and receiving pharmacological treatment 60% had their BP controlled. Hypertension control among all hypertensive participants was little more than one tenth (12.2%, 95% CI: 6.3%-18.1%).

**Table 4:** Distribution of Non-pharmacological approaches to the management of hypertension among Kaya HDSS adult population

Non-pharmacological management of hypertension	Aware who received advice (n=28 out of 33)		Treated pharmacologically who received advice (n=23 out of 25)	
	%	95% CI	%	95% CI
Sodium reduce	96.4	89.1-100.0	95.7	86.6-100.0
Weight loss	32.1	13.7-50.6	34.8	13.7-55.8
Quit smoking	25.0	7.9-42.1	30.4	10.1-50.8
Physical exercise	35.7	16.8-54.8	43.5	21.6-65.4
Alcohol reduce	35.7	16.8-54.6	34.8	13.7-55.8

**Figure 2:**
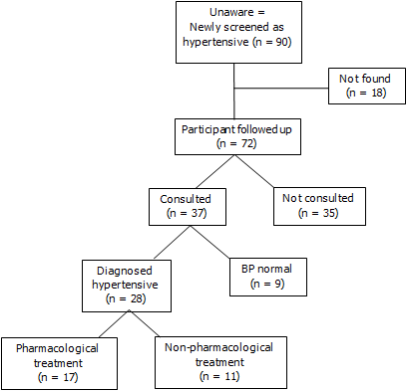
Results of 2 months follow-up of those who had elevated figures of blood pressure in the original hypertension survey and who were unaware of their possible hypertension in Kaya HDSS, Burkina Faso

Of the 90 individuals who were unaware of their hypertensive during the survey, after at least 2 attempts, 72 were located for an interview two months later, 18 were away on the day of our follow up visit. Of those 72 individuals, 37 (51.4%, 95% CI: 39.9%-63.0%) had consulted a nurse and 35 (48.6%) had not consulted any health professional. Reasons given for not consulting a health care professional were: lack of money, lack of time and long distance to the health center. Of the 37 participants who consulted, 9 (24.3%, 95% CI: 10.5%-38.1%) had normal blood pressure and 28 (75.7%, 95% CI: 61.9%-89.5%) were diagnosed with hypertension; of these, 17 (60.7%, 95% CI: 42.3%-78.8%) received medication prescription (Figure 2).

## Discussion

Our results show that in the North-Central region of Burkina Faso, BP measuring was not common. The proportion of adults who had never measured their BP (2 out of 5 adults) is high and comparable to that found among adults (54%) in Northern Angola [9]. Ignorance of the population and inaccessibility of health services could explain that situation and one of its consequences will be a low awareness of hypertension. Accordingly, in this region of Burkina Faso, awareness of hypertension was low and comparable to some countries in West Africa [19] and elsewhere in African countries [9, 20]. Awareness hardly exceeds 40% in sub-Saharan Africa [6, 10]. In developed countries, detection is higher; on the average 49% for men and 62% for women [21].

The low awareness in African countries results from a combination of lack of knowledge about hypertension, lack of availability of primary care and missed opportunities for diagnosis in primary care [22, 23]. In this study only 37% of our participants had knowledge about hypertension, similar levels of ignorance were reported in Ouagadougou [11] and Nigeria [23]. Lack of primary health care including blood pressure screening has been found elsewhere in Africa [9]. Because hypertension is mostly asymptomatic, it is usually not detected without actively seeking attention. Blood pressure measurement is offered in health facilities and pharmacies but payments under the counter are common. Pharmacies often charge about 1 dollar US for the service.

Presence of a chronic disease, semi-urban areas and physical inactivity were associated with higher awareness. Detection seems to involve persons who are more likely to have had encounters with a health care professional who could have diagnosed the hypertension: these are individuals with additional health issues or living in a stressed environment. So people who stated having good health (without symptoms) were less likely to have their blood pressure taken. It is therefore important to educate health professionals to provide screening for hypertension in primary care settings. Living in the semi-urban area was associated with increased awareness of hypertension. This finding corroborates previous results in Africa [8, 19]. This is probably due to better equipment and better access to health services in semi-urban areas compared to rural areas.

Burkina Faso is a developing country where 43.9% of the households lives below the poverty threshold (less than one dollar US a day) [24]. However, in Kaya HDSS, the proportion of treated patients is among the highest in sub-Saharan Africa and close to the highest reported in a study from Ghana, where 82% of aware hypertensive patients were on treatment [7]. Result of treatment of other studies were between 13.9% and 52% of those who were aware [8, 9, 19, 20]. In our study, the fact that 75% of those who were aware were on treatment could be explained by their additionally poor health status. The proportion of aware hypertensive that was not on drug treatment was rather significant (25%). The proportion of treated hypertensive patients with controlled blood pressure in this study was higher (60%) than that found in other African countries [6, 25, 26].

Calcium channel blocker (CCB) was the most widely used anti hypertension medicine in monotherapy as found in Cote d'Ivoire [27]. This was in line with European society of hypertension and European society of cardiology guidelines of hypertension management which recognized five major classes of antihypertensive agents (Thiazide diuretics, CCBs, ACEIs, Angiotensin receptor blocker (ARBs), β-blockers (BB)) as suitable for initiative treatment [18, 28]. WHO prefers Diuretics or CCB instead of BB or ACEI, in monotherapy for afro-american patients [29]. This predominance of CCB does not comply with the Seventh Report of the Joint National Committee on Prevention, Detection, Evaluation, and Treatment of High Blood Pressure (JNC 7) guidelines which recommended thiazide-type diuretics as initial therapy for most patients. But selection of one of 4 other classes (ACEIs, ARBs, CCBs, BBs) as initial therapy was recommended when a diuretic cannot be used or when a compelling indication is present [17].

The follow-up results showed a low utilization of health care. Despite our advice, nearly half did not seek medical attention. This low attendance of health facilities has been previously recognized in Tanzania [22]. It could indicate ignorance or more likely, a lack of resources. In Burkina Faso, public health insurance is not available and patients must pay for care out of their pockets. Lack of transportation and time because of work and home duties may also discourage people from obtaining further medical attention.

### Strengths and limitations

To our knowledge, a study on the awareness, treatment and control has not yet been published on the semi-urban and rural population of Burkina Faso; the present study is the first of its kind and results are based on a randomly selected sample which was representative of the population. Also this is the first study that shows the results of follow up of newly screened hypertensive in West Africa. This study has limitations. The sample size is small and we had insufficient statistical power to analyze factors associated with treatment and control among those who were aware of their condition.

## Conclusion

In this rural and semi-urban population, hypertension is largely unknown among the population. Blood pressure screening is not widespread among the population. As a result, awareness is low, and probably only concentrated in the sicker patients with other chronic conditions. In Kaya HDSS, the proportion of treated patients among those aware is relatively high, at least when compared to other populations in West Africa. The efficacy of hypertension treatment is good because a relatively high proportion of treated patients had their blood pressure under control.
